# Vaginal Microbiota in Women With Spontaneous Preterm Labor Compared to Spontaneous Term Labor: A Cross-Sectional Analytical Study

**DOI:** 10.7759/cureus.82302

**Published:** 2025-04-15

**Authors:** E. Vinodhini, Bhabani Pegu, Sasirekha Rengaraj, Rakesh Singh

**Affiliations:** 1 Department of Obstetrics and Gynecology, Jawaharlal Institute of Postgraduate Medical Education and Research, Puducherry, IND; 2 Department of Microbiology, Jawaharlal Institute of Postgraduate Medical Education and Research, Puducherry, IND

**Keywords:** antibiotic sensitivity pattern, microorganism, preterm labor, spontaneous labor, vaginal infection

## Abstract

Background: Preterm birth is the second leading cause of neonatal morbidity and mortality. This study aimed to determine if there were differences in the microbiota between women experiencing preterm labor and those with full-term pregnancies. Additionally, we aimed to identify the vaginal microorganisms present and their antibiotic sensitivities in women undergoing spontaneous preterm labor.

Methods: Women with a spontaneous onset of labor were recruited for the study. Those with spontaneous term labor were classified as Group A, while those with preterm labor were classified as Group B. Three high vaginal swabs were collected from the posterior fornix using a sterile swab and sent to the Microbiology Department for Gram staining, culture and sensitivity testing, and polymerase chain reaction analysis.

Results: The frequency of vaginal infection is more among women in spontaneous preterm labor than those in spontaneous term labor. Socioeconomic status and women with a previous history of preterm labor were found to be at high risk for preterm labor. The most common organism belongs to Gram-negative bacilli and is sensitive to third-generation cephalosporins.

Conclusion: The study suggests that vaginal infections may increase the risk of preterm labor, with gram-negative bacilli commonly identified and mostly sensitive to third-generation cephalosporins. Routine screening for asymptomatic genital infections in antenatal women is recommended, regardless of preterm labor risk, as early identification and treatment could help prevent preterm labor.

## Introduction

Preterm birth is the leading cause of neonatal mortality and the second most common cause of death in children under five. Each year, 15 million babies are born prematurely, and 85% of neonatal deaths occur in preterm infants [1]. Globally, the prevalence of preterm birth is 10.6%, with South Asia contributing to over a third of these cases. Additionally, nearly three million stillbirths occur annually, 98% of which take place in developing countries [2]. Preterm births can result in immediate complications such as respiratory distress syndrome, sepsis, and intraventricular hemorrhage, as well as long-term issues including asthma, developmental delays, and an increased risk of chronic diseases [3-5]. Various organisms have been associated with preterm birth, chorioamnionitis, and early-onset neonatal sepsis, including *Gardnerella vaginalis*, *Ureaplasma urealyticum*, *Mycoplasma hominis*, *Chlamydia trachomatis*, *Trichomonas vaginalis*, *Neisseria gonorrhoeae*, Actinomyces, and Candida species. These organisms account for 40% of unexplained preterm labor cases, with women affected by these infections showing greater diversity in their vaginal microbiota [6]. This microbial diversity may trigger preterm labor by activating inflammatory pathways crucial to childbirth [7-11]. Early detection and treatment of these infections can help mitigate associated risks.

There are limited studies from India regarding the prevalence and antibiotic sensitivity patterns of vaginal microorganisms in women with preterm labor [12]. Given the changing patient demographics and evolving microbial profiles, further research is essential to better address preterm labor associated with vaginal infections. Antimicrobial stewardship is a continuous process that requires ongoing research to monitor shifts in vaginal microorganism profiles and antibiotic resistance patterns. The selection of empirical antibiotics should be informed by location-specific and hospital-specific data. The study aimed to explore whether there were differences in the microbiota between women experiencing preterm labor and those with full-term pregnancies, as well as to identify the vaginal microorganisms present and their antibiotic sensitivities in women undergoing spontaneous preterm labor.

## Materials and methods

Study design and setting

This study was conducted at the Department of Obstetrics and Gynecology of a tertiary care teaching hospital from August 2021 to July 2023. A prospective, observational design was employed to compare vaginal infections in women with spontaneous preterm labor (Group B) and spontaneous term labor (Group A).

Study population

The study included pregnant women over the age of 18 with established labor. Women were eligible if they met the following criteria: a singleton pregnancy, confirmed gestational age, and either spontaneous preterm labor (defined as <37 weeks of gestation) or spontaneous term labor (≥37 weeks of gestation).

Exclusion criteria

Women were excluded from participation if they had any identifiable fetal risk factors, such as multiple gestation, polyhydramnios, fetal anomalies, or a prolonged rupture of membranes (ROM) lasting more than 18 hours. Additional exclusion criteria included recent use of antibiotics (within the past 14 days), known infections (e.g., urinary tract infections or systemic infections), and those with other comorbidities that could affect the results.

Sampling and recruitment

Written informed consent was obtained from all eligible women who agreed to participate in the study. Women with spontaneous term labor (Group A) were consecutively recruited following the delivery of each spontaneous preterm labor case until the target sample size was achieved.

Data collection

A predesigned proforma was utilized to collect relevant sociodemographic and clinical data, including maternal age, education, occupation, parity, gestational age at delivery, duration of labor pain, duration of ROM or premature ROM, history of fever, vaginal discharge, and estimated fetal weight.

Sample collection and microbiological analysis

Under sterile, aseptic conditions, each participant was positioned in the lithotomy position, and the posterior vaginal wall was retracted using a Sims speculum. Vaginal pH was assessed by applying a pH strip directly to the vaginal mucosa, and the color change was used to classify the pH as either acidic or alkaline.

Three high vaginal swabs were collected from the posterior fornix using sterile swabs. These were subsequently sent to the Microbiology Department for analysis, which included the following.

1. Gram staining: The first swab was smeared onto a glass slide, Gram-stained, and examined microscopically for bacterial morphology and other relevant features.

2. Culture and sensitivity testing: The second swab was inoculated on both blood agar and MacConkey agar plates, incubated at 37°C for 24 hours, and assessed for bacterial growth. Aerobic and facultative anaerobic bacteria, as well as yeast isolates, were identified using standard microbiological methods. The antibiotic sensitivity testing was conducted using the disk diffusion method, following the guidelines set by the Clinical and Laboratory Standards Institute.

3. Rapid antigen testing and polymerase chain reaction: The third swab was used for *T. vaginalis* detection via rapid antigen testing, while polymerase chain reaction was performed to detect *C. trachomatis* and *M. hominis*. In vitro antibiotic susceptibility testing was conducted using an automated method.

Sample size calculation

**Figure 1:**
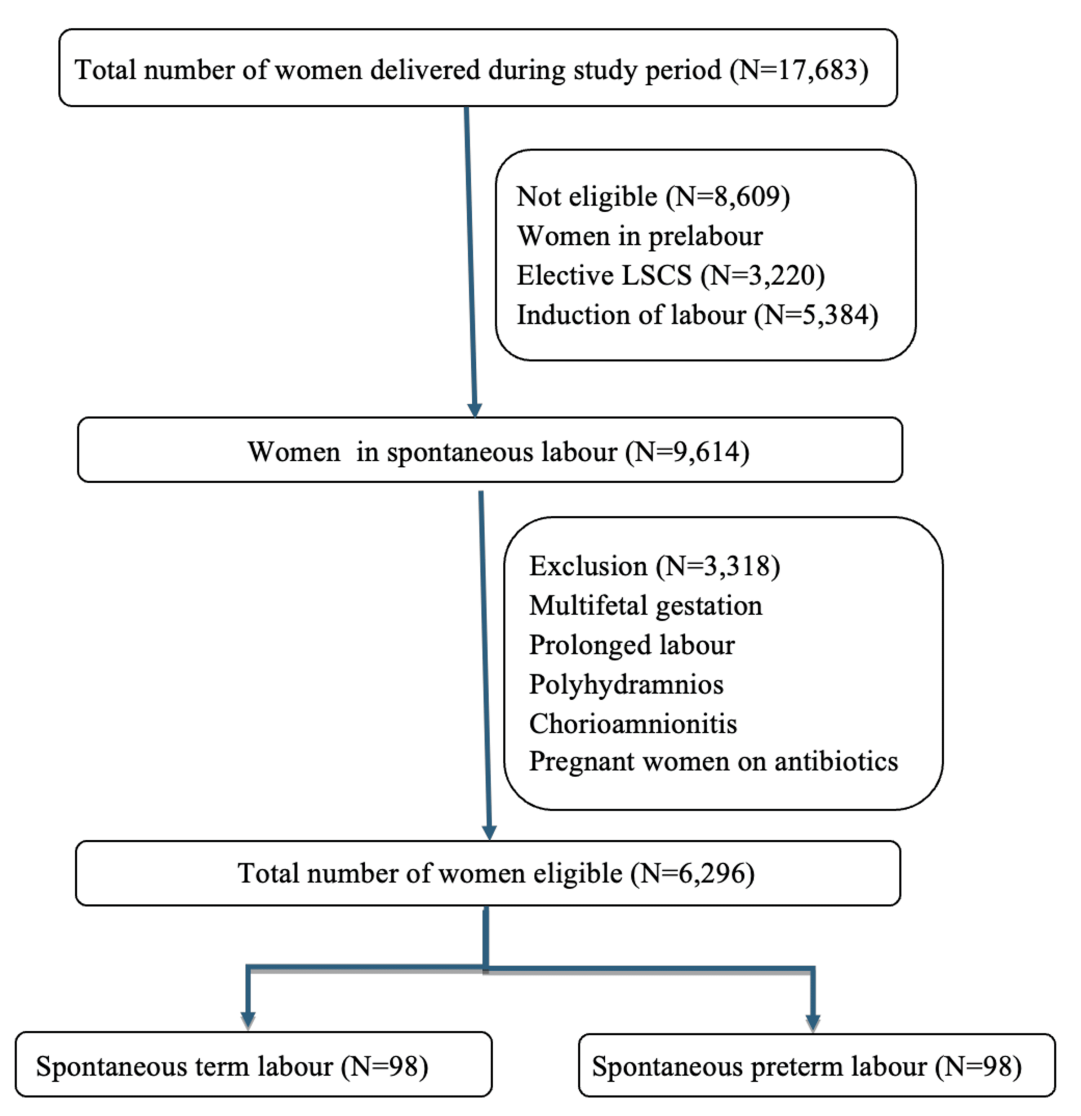
Flowchart for case enrolment

The sample size was determined based on literature reporting vaginal infection rates of 28% in women with preterm labor and 10% in women with term labor [13]. To detect an 18% higher prevalence of vaginal infections in women with spontaneous preterm labor compared to those with spontaneous term labor, with 90% power and a 5% margin of error, a total sample size of 196 women was required (98 in each group). Figure 1 shows the flowchart for case enrolment.

Statistical analysis

Data were analyzed using the Statistical Package for the Social Sciences software (version 30; IBM Corp., Armonk, NY). Categorical variables (e.g., presence of vaginal infections) were expressed as frequencies or proportions. Continuous variables (e.g., maternal age and gestational age) were summarized as means with standard deviations or medians with interquartile ranges, depending on the data distribution. The difference in the proportion of vaginal infections between the two groups was analyzed using the chi-square test or Fisher's exact test, as appropriate.

Ethical approval

This study was conducted in accordance with the ethical standards established by the Institute's Scientific Advisory and Ethics Committee for human research, adhering to the principles outlined in the 1964 Declaration of Helsinki and its subsequent revisions. The study received approval from the Ethics Committee of the Jawaharlal Institute of Postgraduate Medical Education and Research for human studies (approval number: JIP/IEC/2021/239; dated August 6, 2021).

## Results

Among the 98 women in spontaneous term labor in Group A, 41 (41.8%) had vaginal infections with pathogenic microorganisms, while 57 (58.2%) had normal vaginal flora. In Group B, consisting of 98 women in spontaneous preterm labor, 69 (70.4%) had vaginal infections and 29 (29.6%) had normal vaginal flora. This difference was statistically significant with a p value of 0.001, as determined by the chi-square test. A comparison of baseline characteristics and clinical parameters of both groups is shown in Table 1.

**Table 1:** Demographic data, risk factors, and clinical profiles of women with spontaneous preterm and term deliveries BMI: body mass index; GDM: gestational diabetes mellitus; DM: diabetes mellitus ^*^Chi-square test

Parameters		Group A (n = 98)	Group B (n = 98)	p value^*^
Age (years), median (interquartile range)		24 (22-27)	25 (23-28)	0.102
Socioeconomic status, n (%)	Upper middle	6 (6.12)	15 (15.31)	0.021
	Middle	-	3 (3.06)	
	Lower middle	92 (93.8)	80 981.63)	
Education status, n (%)	Primary school	5 (5.1)	15 (15.31)	0.271
	High school	65 (66.3)	50 (51)	
	Graduate	28 (28.57)	33 (33.6)	
BMI (kg/m^2^), median (interquartile range)		22.3 (21.3-24.2)	23.2 (22-25.3)	0.129
BMI (kg/m^2^), n (%)	<18.5	-	-	
	18.5-24.9	80 (81.63)	70 (71.43)	
	25-29.9	18 (18.37)	28 (28.57)	
	≥30	-	-	
Hemoglobin, n (%)	≥11 g/dL	55 (56.12)	45 (45.9)	0.198
	<11 g/dL	43 (43.88)	53 (54)	
Diabetes, n (%)	GDMA1	10 (83.3)	11 (42.3)	0.094
	GDMA2	2 (16.7)	9 (34.6)	
	Type 2 DM	-	5 (19.2)	
	Type 1 DM	-	1 (3.8)	
Gravida, n (%)	Primigravida	57 (58.16)	54 (55.1)	0.773
	Multigravida	41 (41.84)	44 (44.9)	
History of preterm labor, n (%)	Present	-	7 (7.14)	0.014
	Absent	98 (100)	91 (92.86)	
Premature rupture of membranes, n (%)	Present	17 (17.35)	6 (6.12)	0.251
	Absent	81 (82.65)	92 (93.88)	

Socioeconomic status and a history of previous preterm labor were found to significantly impact the spontaneous onset of preterm labor, with statistically significant differences between the two groups. Other factors, such as age, BMI, comorbidities, and parity, were comparable between the groups.

Of the 196 parturient with spontaneous labor, 110 (56.12%) were diagnosed with vaginal infections. Among these, 82 women had an acidic vaginal pH, while 28 women had an alkaline vaginal pH. The most common pathogens identified in the preterm labor group were *Escherichia coli*, followed by *Klebsiella pneumoniae*, Pseudomonas species, and Candida species. The distribution of vaginal microbiota in women with preterm and term labor is presented in Table 2.

**Table 2:** Proportion of vaginal microflora in women with preterm and term labor

Vaginal microorganisms	Proportion of women in preterm labor, n (%)	Proportion of women in term labor, n (%)
Normal flora	29 (2.6)	57 (58.2)
Chlamydial infection	1 (7.1)	2 (13.3)
Mycoplasma infection	13 (92.9)	10 (66.7)
E. coli	18 (18.37)	12 (12.24)
K. pneumoniae	10 (10.2)	4 (4.08)
Pseudomonas species	6 (6.12)	3 (3.06)
Enterobacter species	3 (3.06)	3 (3.06)
Acinetobacter species	2 (2.04)	1 (1.02)
Enterococcus faecalis	2 (2.04)	-
Candida species	23 (23.47)	28 (21.42)

Most of the identified organisms were found to be sensitive to third-generation cephalosporins, as shown in Table 3.

**Table 3:** Antibiotic sensitivity of the common vaginal microorganisms

Antibiotic tested	Number of sensitive *E. coli* (n = 30)	Number of sensitive *K. pneumoniae* (n = 10)
Ceftriaxone	27	9
Ceftazidime	30	10
Cefoperazone and sulbactam	30	10
Ciprofloxacin	30	10
Piperacillin and tazobactam	30	10
Amikacin	24	10

## Discussion

Our study explored the factors contributing to spontaneous preterm labor, providing valuable insights into the connection between vaginal infections, maternal health conditions, and the timing of preterm birth.

Among the women with spontaneous preterm labor, 70.4% (69 women) had vaginal infections, compared to 41.8% (41 women) in the term labor group. Aside from socioeconomic status and a history of previous preterm labor, factors such as age, BMI, comorbidities, and parity were comparable between the two groups. Most preterm births occurred between 33 and 35 weeks of gestation, which accounted for approximately 62% of all preterm cases. The most common pathogens isolated in the preterm labor group were *E. coli*, followed by *K. pneumoniae*, Pseudomonas species, and Candida species. Interestingly, most of these organisms demonstrated sensitivity to third-generation cephalosporins, such as ceftriaxone and ceftazidime.

The risk of preterm labor is notably increased in women with a history of previous preterm births. This finding aligns with existing literature, which highlights that women with a history of spontaneous preterm labor are at a higher risk of subsequent preterm deliveries [14,15]. One study reported that 20.3% of women with a prior preterm birth experienced recurrent preterm singleton births [16]. Our study also examined the role of anemia in preterm labor and found no significant association. This is consistent with previous studies suggesting that anemia in the first trimester may increase the risk of preterm birth. In contrast, anemia in later trimesters does not correlate with preterm labor. However, a 2016 meta-analysis suggested that anemia during pregnancy contributes to 19% of preterm births [17].

In terms of maternal diabetes, our study noted a higher prevalence of gestational and pregestational diabetes in the preterm labor group, but no significant correlation between diabetes and preterm birth was observed. These findings are consistent with a study that found no significant difference in pregnancy outcomes, including preterm labor, between women with and without gestational diabetes [18]. However, other studies have recognized both gestational and pregestational diabetes as independent risk factors for preterm birth [19].

Our study also examined the relationship between vaginal infections and vaginal pH. Women without vaginal infections maintained an acidic vaginal pH, while those with vaginal infections had a higher vaginal pH. Specifically, 25.5% of women with vaginal infections had elevated pH levels compared to only 5.8% in the noninfected group. This supports recent research showing that vaginal dysbiosis, characterized by an elevated pH, is a significant risk factor for preterm labor [14]. An acidic vaginal pH helps protect against dysbiosis, and any increase in pH could lead to the acquisition of polymicrobial flora, which can contribute to preterm birth. These findings are consistent with studies linking elevated vaginal pH to increased risk of vaginal infections and preterm delivery [20].

When comparing women with preterm and term labor, we found that women in preterm labor were more likely to have vaginal infections. Altered vaginal microbiota, particularly the presence of *G. vaginalis*, has been associated with adverse pregnancy outcomes, including preterm birth, through immune responses and other host factors [21]. Moreover, intra-amniotic infections, often originating from ascending vaginal infections, are a well-established cause of preterm birth [22]. Over time, research has consistently shown that infections through various pathways contribute to preterm labor. The reduction of preterm infant mortality by extending gestational age at birth remains a central goal in neonatology, which can be achieved through effective management of high-risk pregnancies and advancements in neonatal intensive care [23].

The most commonly identified pathogens in women with spontaneous preterm labor in our study were *E. coli*, *K. pneumoniae*, *Pseudomonas aeruginosa*, and Candida species. Notably, none of the women in either the term or preterm labor groups tested positive for *T. vaginalis* infection, which is relatively uncommon at a tertiary care center due to syndromic management at the grassroots level. Previous studies have suggested potential benefits from adjunct antibiotic therapy in managing preterm labor [24]. In our study, the organisms commonly isolated from vaginal swabs, including *E. coli* and *K. pneumoniae*, showed sensitivity to ceftriaxone, ceftazidime, cefoperazone-sulbactam, ciprofloxacin, and amikacin, indicating that appropriate antimicrobial treatment could play a role in managing infections in women at risk of preterm labor.

This study underscores the importance of early detection and treatment of vaginal infections as well as careful management of maternal health conditions such as diabetes and anemia to potentially reduce the risk of preterm birth.

The strengths of this study include its prospective design, which allows for a thorough examination of various risk factors and the role of vaginal infections in preterm labor. It also provides valuable insights into the vaginal microorganisms prevalent in the local population, aiding the development of targeted, population-specific antibiotic therapies. However, several limitations must be acknowledged. First, the nonspecific differences observed between the two study groups may be attributed to the small sample size. Second, we did not conduct follow-up assessments to evaluate maternal and neonatal outcomes, particularly for women with preterm labor. Finally, due to logistical constraints, only a limited number of organisms were cultured.

## Conclusions

To conclude, the study's findings indicate that vaginal infections may contribute to the risk of preterm labor. Gram-negative bacilli were frequently found in these women, with the majority being sensitive to third-generation cephalosporins. Given these results, it is recommended that routine screening for asymptomatic genital infections be conducted in antenatal women, incorporating both clinical criteria and diagnostic testing, regardless of their preterm labor risk. Early detection and treatment of vaginal infections could play a crucial role in preventing preterm labor.
